# Diagnosis and Management of Bloodstream Infections With Rapid, Multiplexed Molecular Assays

**DOI:** 10.3389/fcimb.2022.859935

**Published:** 2022-03-16

**Authors:** Sherry A. Dunbar, Christopher Gardner, Shubhagata Das

**Affiliations:** Luminex Corporation, A DiaSorin Company, Austin, TX, United States

**Keywords:** bloodstream infection, molecular diagnostics, microarray, antimicrobial stewardship, antibiotic resistance

## Abstract

Bloodstream infection is a major health concern, responsible for considerable morbidity and mortality across the globe. Prompt identification of the responsible pathogen in the early stages of the disease allows clinicians to implement appropriate antibiotic therapy in a timelier manner. Rapid treatment with the correct antibiotic not only improves the chances of patient survival, but also significantly reduces the length of hospital stay and associated healthcare costs. Although culture has been the gold standard and most common method for diagnosis of bloodstream pathogens, it is being enhanced or supplanted with more advanced methods, including molecular tests that can reduce the turnaround time from several days to a few hours. In this article, we describe two rapid, molecular bloodstream infection panels that identify the most common pathogens and associated genetic determinants of antibiotic resistance – the Luminex^®^ VERIGENE^®^ Gram-Positive Blood Culture Test and the VERIGENE^®^ Gram-Negative Blood Culture Test. We conducted a search on PubMed to retrieve articles describing the performance and impact of these tests in the clinical setting. From a total of 48 articles retrieved, we selected 15 for inclusion in this review based on the type and size of the study and so there would be minimum of three articles describing performance and three articles describing the impact post-implementation for each assay. Here we provide a comprehensive review of these publications illustrating the performance and clinical utility of these assays, demonstrating how genotypic tests can benefit diagnostic and antimicrobial stewardship efforts.

## Introduction

Bloodstream infection (BSI) is a serious, life-threatening illness and a major cause of morbidity and mortality worldwide (Santella et al., 2020; Timsit et al., 2020). Estimates have indicated that BSI affects approximately 30 million people each year, leading to 6 million deaths (Wisplinghoff et al., 2004; Fleischmann et al., 2016). The outcome of BSI can depend on host-specific factors (including age and underlying comorbidities) and microbiological aspects (including the type of the infecting organism and antibiotic susceptibility), but some estimates have indicated that the mortality rate can be as high as 25% to 80% (Kollef et al., 2011; Levy et al., 2014). Prompt initiation of suitable antimicrobial therapy is vital and can significantly improve patient management and decrease the mortality rate in bacteremic patients (Leibovici et al., 1998; Kumar et al., 2006).

Gram-positive pathogens are involved in >50% of all bacterial bloodstream infections, and coagulase-negative staphylococci (CoNS), *Staphylococcus aureus*, and *Enterococcus* spp. are the most often isolated gram-positive bacteria in blood cultures (Beekmann et al., 2003; Wisplinghoff et al., 2004; Grozdanovski et al., 2012). At least 30% of hospital-acquired BSIs are due to gram-negative bacteria (Gaynes and Edwards, 2005; Diekema et al., 2019). Infections caused by these pathogens, particularly in hospital-acquired infections, have been linked to a 15% to 29% higher mortality as compared to case controls (Wisplinghoff et al., 2003; Al-Hasan et al., 2012). This is especially true for infections with multidrug-resistant organisms, such as those harboring extended-spectrum β-lactamases (ESBLs) or carbapenemases (Schwaber et al., 2006; Patel et al., 2008; Kaye and Pogue, 2015; Kern and Rieg, 2020).

Although culture with biochemical and susceptibility testing has long been the standard method for diagnosis of bloodstream pathogens, it is being enhanced and, in some cases, supplanted with more advanced methods (Idelevich et al., 2018; Lamy et al., 2020). Among these new and more rapid diagnostic methods are molecular tests that can reduce the turnaround time from several days to a few hours. While implementation of these rapid diagnostics tests may add cost to the clinical microbiology laboratory, they can transform the management of BSI patients by providing antimicrobial stewardship teams the opportunity to quickly escalate therapy and improve patient outcomes through prompt organism identification and detection of resistance determinants, and thus provide a net financial benefit to the institution (Walker et al., 2016).

In this article, we describe two of these rapid molecular bloodstream infection panels, the Luminex^®^ VERIGENE^®^ Gram-Positive Blood Culture Test (BC-GP) and the VERIGENE^®^ Gram-Negative Blood Culture Test (BC-GN), and review some of the key publications that demonstrate the performance and clinical utility of these tests which illustrate how genotypic tests can benefit both diagnostic and antimicrobial stewardship programs. For this review, we conducted a literature search in PubMed^®^ (https://pubmed.ncbi.nlm.nih.gov/) on the product name of each panel. We retrieved a total of 48 citations published between 2013 and 2020. Studies were considered for inclusion if they described a performance evaluation, clinical or economic effects post-implementation, conventional methods were used as comparators, and the research was conducted in a clinical laboratory setting. Publications were then ordered based on the study size or number of samples or subjects included, and selected so that there would be a minimum of three publications on performance and three on clinical/economic impact for each assay. Studies were excluded if the comparator was not an IVD-cleared test. A total of fifteen representative studies have been described for this review.

The VERIGENE Blood Culture Nucleic Acid tests are qualitative, multiplexed *in vitro* diagnostic tests for the simultaneous detection and identification of bacteria which may cause BSI (Luminex, 2021a). The tests are performed directly on media from blood culture bottles that have been identified as positive (by a continuous monitoring blood culture system), using the sample-to-answer VERIGENE^®^ System.

**Figure 1:**
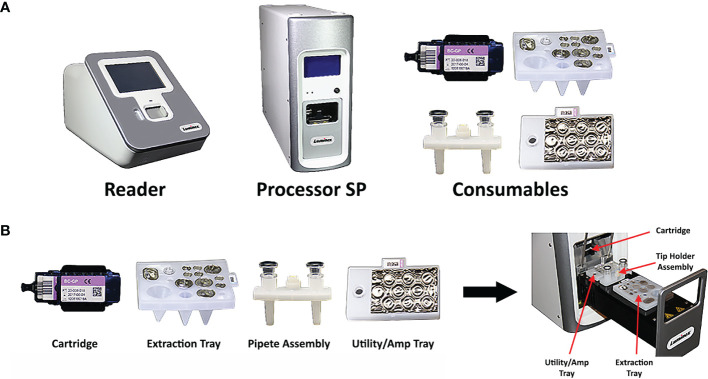
Composition of the VERIGENE^®^ System. **(A)** The VERIGENE System includes the VERIGENE Reader, the Processor SP, and the consumables for the specific assay. **(B)** The VERIGENE test consumables include the VERIGENE Test Cartridge, the extraction tray, the pipette assembly, and the utility/amp tray.

The VERIGENE System is a moderately complex sample-to-answer molecular diagnostic instrument for direct or post-PCR detection of specific nucleic acid targets (Luminex, 2021b). The system is comprised of the VERIGENE Reader (reader), the Processor SP (sample processor), and the consumables for the specific assay, which include the VERIGENE Test Cartridge (test cartridge), extraction tray, pipette assembly, and utility/amp tray (**Figure 1**). The reader can be connected to either one or multiple processors. The reader handles sample information, reads the results from test cartridges, and allows the results to be printed, and also provides internal data storage and laboratory information system (LIS) connectivity without the need for an external computer. The processor is a modular, benchtop analysis unit that conducts automated nucleic acid extraction, purification, amplification, and hybridization in each module. The system allows users to run tests as needed in response to testing requirements, without the need for batching, and it is scalable to allow laboratories to customize the throughput to meet their specific needs. The system features a simple user interface and does not require extensive training or specialized facilities. The test cartridges are self-contained, single-use units consisting of a microfluidic cassette containing the hybridization reagents which also captures the waste generated during test processing (the Reagent Pack), and a glass slide as the solid support for the microarray that captures the targeted nucleic acids (the Substrate Holder). Each test cartridge can be used for analysis of one patient sample.

The VERIGENE NanoGrid™ Technology is a novel, proprietary gold nanoparticle probe chemistry that is used for all VERIGENE tests. The gold nanoparticles are 13-20 nanometers (nm) in diameter and can be functionalized with either a defined number of oligonucleotides (oligos) specific for a DNA or RNA target of interest, or a defined number of antibodies specific to a protein of interest. Gold nanoparticle probes can allow for sensitivity that is several orders of magnitude higher as compared to fluorophores (Huang and El-Sayed, 2010). They also enable high specificity for nucleic acid or protein detection and reduce background noise, which creates an enhanced assay signal through an improved signal-to-noise ratio. Gold nanoparticle probes are also stable with a long shelf-life and are non-toxic.

**Figure 2:**
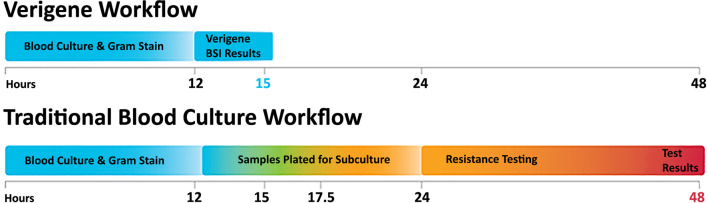
The VERIGENE^®^ workflow as compared to traditional blood culture. Positive blood cultures are evaluated by Gram stain and the appropriate VERIGENE Blood Culture Nucleic Acid Test is selected. Set-up, running the test, and reporting can be easily completed within 3 hours. With traditional culture-based workflows, subculture can add an additional 12 hours, and resistance testing (when required) can add another day so that the final results may not be available for 48 hours.

The detection of nucleic acid targets on the VERIGENE System includes: 1) automated nucleic acid extraction and fragmentation (or PCR amplification) from a clinical sample on the processor; 2) automated transfer of the processed nucleic acids into a test cartridge for hybridization; 3) primary hybridization of the target DNA to capture oligos on the microarray; 4) secondary hybridization of specific mediator oligos and gold nanoparticle probes; 5) signal amplification of hybridized probes through silver staining; and 6) automated qualitative reading and analysis of the results on the reader. The time to result from sample input to result is less than two hours. An overview of the workflow as compared to conventional culture methods is shown in **Figure 2**.

Two separate tests are available, for either gram-positive or gram-negative bacteria, and the appropriate test is selected based on the type(s) of bacteria present in the positive blood culture bottle, as determined by Gram stain. The BC-GP and BC-GN tests detect and identify the bacterial species, genera, and resistance markers as shown in **Tables 1** and **2**, respectively. The VERIGENE bloodstream infection tests allow laboratories to quickly identify bacterial pathogens, as well as their resistance markers. Published studies have shown that these assays lower overall hospital costs, shorten a patient’s length of hospital stay, and improve patient outcomes by supporting a more targeted treatment plan quicker than traditional methods (Rivard et al., 2017). The assays have also been shown to coordinate well with antimicrobial stewardship programs (ASPs), decrease the use of unnecessary antibiotics, and efficiently help manage infection control. (Box et al., 2015; Rivard et al., 2017). A review on cost-effectiveness of rapid diagnostic tests for BSIs found that implementation of BC-GP and BC-GN with an ASP was associated with lower costs than baseline strategy and is a cost-effective approach, although sensitivity analysis showed that the effectiveness is dependent on the probability of mortality or survival (Pliakos et al., 2018).

**Table 1:** Targets included in the VERIGENE Gram-Positive Blood Culture test.

Species	Genus	Group	Resistance
*Staphylococcus aureus*	*Staphylococcus* spp.	*Streptococcus anginosus*	*mecA* (methicillin)
*Staphylococcus epidermidis*	*Streptococcus* spp.		*vanA* (vancomycin)
*Staphylococcus lugdunensis*	*Micrococcus* spp.^a^		*vanB* (vancomycin)
*Streptococcus agalactiae*	*Listeria* spp.		
*Streptococcus pneumoniae*			
*Streptococcus pyogenes*			
*Enterococcus faecalis*			
*Enterococcus faecium*			

a Micrococcus spp. is not IVD-cleared in the United States.

**Table 2:** Targets included in the VERIGENE Gram-Negative Blood Culture test.

Species	Genus	Resistance
*Escherichia coli* ^a^	*Acinetobacter* spp.	CTX-M (ESBL)
*Klebsiella pneumoniae*	*Citrobacter* spp.	IMP (carbapenemase)
*Klebsiella oxytoca*	*Enterobacter* spp.	KPC (carbapenemase)
*Pseudomonas aeruginosa*	*Proteus* spp.	NDM (carbapenemase)
*Serratia marcescens* ^b^		OXA (carbapenemase)
		VIM (carbapenemase)

a Test does not distinguish Escherichia coli from Shigella spp. (S. dysenteriae, S. flexneri, S. boydii, and S. sonnei).

b Serratia marcescens is not IVD-cleared in the United States.

## Performance

### The Luminex^®^ VERIGENE^®^ Gram-Positive Blood Culture Test

The clinical performance of the VERIGENE BC-GP test was established in a study conducted at five hospital sites geographically distributed across the United States (Luminex, 2020c). A total of 1642 specimens were included in the comparison, comprised of 1426 prospectively collected specimens (1251 fresh and 175 frozen) and 216 simulated specimens utilized for organisms not frequently found in the prospective specimens (i.e., *Streptococcus lugdunensis*, *Streptococcus agalactiae*, *Streptococcus anginosus* group, *Streptococcus pyogenes*, and *Listeria* spp.). The simulated specimens were prepared from glycerol stocks grown on blood agar plates and individual colonies were used to inoculate standard aerobic blood culture media bottles containing whole blood. Blood culture specimens positive by culture using either BACTEC™ Plus Aerobic/F or BacT/ALERT FA FAN^®^ Aerobic blood culture bottles and identified as gram-positive were then processed according to conventional biochemical, culture, and bidirectional sequencing identification methods as the reference comparators. The positive percent agreement (PPA), negative percent agreement (NPA), and the two-sided 95% confidence limits (95% CI) observed for detection of each organism and resistance marker (i.e., *mecA* detection with *Staphylococcus aureus* and *Staphylococcus epidermidis* and *vanA*/*vanB* detection with *Enterococcus faecalis* and *Enterococcus faecium*) are shown in **Table 3**. Other *Staphylococcus* spp. were not tested for methicillin resistance by cefoxitin disk; however, 12 tested positive for *S. epidermidis* and *mecA* by BC-GP and were counted as false positives for *mecA*. Altogether, there were 98 mixed specimens of which 6 were detected by BC-GP only, 25 were detected by the reference method(s) only, and 67 were detected by both. Overall, the test was shown to be highly sensitive and specific with the total PPA ≥93.1% in all cases except for *vanA* linked with *E. faecalis*, which was 85.7%. The total NPA was >98.2% for all targets, with the exceptions of *vanA* linked with *E. faecium* (93.0%) and *mecA* linked with *S. epidermidis* (81.5%).

**Table 3:** Summary of clinical performance for the VERIGENE Gram-Positive Blood Culture test.

Target	Fresh		Frozen		Total		Simulated		
	PPA ^a^N ^b^(95% CI ^c^)	NPA ^d^N (95% CI)	PPAN (95% CI)	NPAN (95% CI)	PPAN (95% CI)	NPAN (95% CI)	PPAN (95% CI)		NPAN (95% CI)
*Staphylococcus aureus*	99.1% 322/325 (97.3-99.8)	100% 926/926 (99.6-100)	100% 10/10 (69.2-100)	100% 165/165 (97.8-100)	99.1% 332/335 (97.4-99.8)	100% 1091/1091 (99.7-100)	NA^e^		100% 216/216 (98.3-100)
*Staphylococcus epidermidis*	93.0% 294/316 (89.6-95.6)	98.7% 923/935 (97.8-99.3)	100% 2/2 (15.8-100)	100% 173/173 (97.9-100)	93.1% 296/318 (89.7-95.6)	98.9% 1096/1108 (98.1-99.4)	100% 2/2 (15.8-100)		100% 214/214 (98.3-100)
*Staphylococcus lugdunensis*	87.5% 7/8 (47.4-99.7)	100% 1243/1243 (99.7-100)	100% 12/12 (73.5-100)	100% 163/163 (97.8-100)	95.0%(h) 19/20 (75.1-99.9)	100% 1406/1406 (99.7-99.9)	100% 20/20 (83.2-100)		99.5% 195/196 (97.2-99.9)
*Staphylococcus* spp.	97.9% 895/914 (96.8-98.7)	99.4% 335/337 (97.9-99.9)	100% 30/30 (88.4-100)	99.3% 144/145 (96.2-99.9)	98.0% 925/944 (96.9-98.8)	99.4% 479/482 (98.2-99.9)	100% 25/25 (86.3-100)		100% 191/191 (98.1-100)
*Listeria* spp.	100% 3/3 (29.2-100)	100% 1248/1248 (99.7-100)	NA	100% 175/175 (97.9-100)	100% 3/3 (29.2-100)	100% 1423/1423 (99.7-100)	100% 34/34 (89.7-100)		100% 182/182 (98.0-100)
*mecA*	94.1% 366/389 (91.2-96.2)	97.8% 843/862 (96.6-98.7)	100% 9/9 (66.4-100)	100% 166/166 (97.8-100)	94.2% 375/398 (91.5-96.3)	98.2% 1009/1028 (97.1-98.9)	NA		100% 216/216 (98.3-100)
*mecA* linked with *S. aureus*	–	–	–	–	97.5% 157/161 (93.8-99.3)	98.8% 172/174(e) (95.9-99.9)	–		–
*mecA* linked with *S. epidermidis*	–	–	–	–	92.0% 219/238 (87.8-95.1)	81.5% 75/92 (72.1-88.9)	–		–
*Enterococcus faecalis*	96.0% 72/75 (88.8-99.2)	99.9% 1175/1176 (99.5-99.9)	100% 21/21 (83.9-100)	100% 154/154 (97.6-100)	96.9% 93/96 (91.1-99.4)	99.9% 1329/1330 (99.6-99.9)	92.3% 12/13 (64.0-99.8)		100% 203/203 (98.2-100)
*Enterococcus faecium*	94.4% 34/36 (81.3-99.3)	100% 1215/1215 (99.7-100)	100% 32/32 (89.1-100)	100% 143/143 (97.5-100)	97.1% 66/68 (89.8-99.6)	100% 1358/1358 (99.7-100)	100% 46/46 (92.3-100)		99.4% 169/170 (96.8-99.9)
*vanA*	91.9% 34/37 (78.1-98.3)	100% 1214/1214 (99.7-100)	96.9% 31/32 (83.8-99.9)	97.9% 140/143 (94.0-99.6)	94.2% 65/69 (85.8-98.4)	99.8% 1354/1357 (99.4-99.9)	100% 15/15 (78.2-100)		100% 201/201 (98.2-100)
*vanB*	–	100% 1251/1251 (99.7-100)	100% 3/3 (29.2-100)	100% 172/172 (97.9-100)	100% 3/3 (29.2-100)	100% 1423/1423 (99.7-100)	97.3% 36/37 (85.8-99.9)		100% 179/179 (98.0-100)
*vanA* linked with *E. faecalis*	–	–	–	–	85.7% 12/14 (57.2-98.2)	100% 95/95 (96.2-100)	–		–
*vanA* linked with *E. faecium*	–	–	–	–	97.2% 69/71 (90.2-99.7)	93.0% 40/43 (80.9-98.5)	–		–
*vanB* linked with *E. faecalis*	–	–	–	–	100% 7/7 (59.0-100)	100% 102/102 (96.5-100)	–		–
*vanB* linked with *E. faecium*	–	–	–	–	97.0% 32/33 (84.2-99.9)	100% 81/81 (95.6-100)	–		–
*S. agalactiae*	97.5% 39/40 (86.8-99.9)	100% 1211/1211 (99.7-100)	100% 31/31 (88.8-100)	100% 144/144 (97.5-100)	98.6% 70/71 (92.4-99.9)	100% 1355/1355 (99.7-100)	100% 6/6 (54.1-100)		100% 210/210 (98.3-100)
*S. pneumoniae*	100% 25/25 (86.3-100)	99.6% 1221/1226 (99.1-99.9)	100% 13/13 (75.3-100)	100% 162/162 (97.8-100)	100% 38/38 (90.8-100)	99.6% 1383/1388 (99.2-99.9)	100% 8/8 (63.1-100)		100% 208/208 (98.2-100)
*S. pyogenes*	100% 10/10 (69.2-100)	100% 1241/1241 (99.7-100)	92.9% 13/14 (66.1-99.8)	100% 161/161 (97.7-100)	95.8% 23/24 (78.9-99.9)	100% 1402/1402 (99.7-100)	98.2% 53/54 (90.1-99.9)		100% 162/162 (97.8-100)
*S. anginosus* group	100% 9/9 (66.4-100)	99.8% 1239/1242 (99.3-99.9)	100% 3/3 (29.2-100)	100% 172/172 (97.9-100)	100% 12/12 (73.5-100)	99.8% 1411/1414 (99.4-99.9)	100% 23/23 (85.2-100)		99.5% 192/193 (97.2-99.9)

a PPA, Positive Percent Agreement.

b N, number.

c 95% CI, 95% Confidence Interval.

d NPA, Negative Percent Agreement.

e NA, not applicable.

Subsequently, in 2014, Mestas et al. at the Children’s Hospital Los Angeles reported the results of a performance evaluation of the BC-GP assay in 203 pediatric patients, as compared to conventional culture-based identification and susceptibility testing (Mestas et al., 2014). Discordant results were resolved by matrix-assisted laser desorption ionization–time of flight mass spectrometry (MALDI-TOF) or 16S rRNA sequencing. The BC-GP assay demonstrated a concordance of 95.8% (206/215). The targets that are included in the BC-GP test covered 96.4% (215/223) of the recovered isolates. Only 3.0% (6/203) of the blood cultures had invalid results (which were related to the internal processing control) and required repeat testing during the study period, and five of these were correctly identified after one repeat, resulting in an overall correct identification rate of 97.0%. The BC-GP assay showed high accuracy in the detection of the *mecA* gene in *S. aureus* and *S. epidermidis*, and correctly identified 100% of methicillin-resistant *S. aureus* (MRSA) and 98% of methicillin-resistant *S. epidermidis* (MRSE), respectively. All vancomycin-resistant *E. faecium* isolates were also correctly identified but no vancomycin-resistant *E. faecalis* isolates were isolated during the study period. One limitation they describe in the study is that the test is unable to differentiate between monomicrobial or polymicrobial cultures containing *Staphylococcus* spp. other than *S. aureus, S. lugdunensis*, and *S. epidermidis*, or containing *Streptococcus* spp. other than *S. pneumoniae*, *S. pyogenes*, *S. agalactiae*, and *S. anginosus* group. And while multiple organisms could not be determined exclusively from the BC-GP results in 25% of cases, the test was able to correctly detect as least one organism from polymicrobial blood cultures in 93.8% of cases, and accurately identify all targeted bacteria and resistance markers in 81.3% of cases.

To evaluate improvements in turnaround time, blood cultures found positive for gram-positive cocci were routinely reflexed to BC-GP testing, which was run 24 hours a day, 7 days a week. The turnaround times for identification of the targeted bacteria were compared in a 4-month pre-implementation period for conventional testing, and a 4-month period post-implementation of BC-GP. Additionally, the turnaround times for reporting susceptibility results for *S. aureus*, *S. epidermidis*, *E. faecalis*, and *E. faecium* compared to detection of resistance genes was conducted for the same time periods. Statistically significant differences in turnaround times between the BC-GP and the conventional methods for identification and resistance detection were calculated using a paired *t* test. Pre-implementation, the average turnaround times for traditional identification and susceptibility reporting were 34.2 hours and 41.4 hours, respectively. After implementation of the BC-GP assay, the average turnaround time was decreased to 4.1 hours for identification and detection of resistance genes. The mean turnaround time for organism identification decreased by 30.1 hours (*P*<0.0001) and by 37.3 hours (*P*<0.0001) for resistance marker detection.

The study by Mestas et al. demonstrates that the BC-GP test is highly accurate for identification of gram-positive bacteria and detection of resistance markers, and strong as compared to the routine laboratory methods. Furthermore, successful integration of the test into the routine workflow of the microbiology laboratory resulted in a marked improvement in turnaround times, especially for identification of MRSA and vancomycin-resistant enterococci (VRE).

The same institution published a more comprehensive study in 2018, which included extensive data describing their results over five years after implementation of the BC-GP test (Vareechon et al., 2018). Within five years, a total of 1636 blood culture bottles positive for a gram-positive organism were tested on the BC-GP panel. BC-GP identified gram-positive organisms in 92.9% (1520/1636) of the blood cultures tested. In positive blood cultures, there was a 96.4% (806/834) agreement of BC-GP to the species level. Compared with conventional antimicrobial susceptibility testing (AST), the PPA for BC-GP was 100% for both MRSA (50) and MRSE (365). The NPA for *mecA* detection in MRSA and MRSE was 99.1% (221/223) and 99.2% (120/121), respectively. The PPA and NPA for vancomycin-resistant *E. faecium* in BC-GP was 100%. None of the 84 blood cultures positive for *E. faecium* were positive for *vanA/B*.

The biggest discrepancy observed was a false-positive rate of 43.1% (25/58) for *Streptococcus pneumoniae*. Most of these were identified as *Streptococcus mitis/oralis* (21/25) by conventional testing and this finding was not unexpected since these species share 99% homology at the sequence level. The authors recommended that identification of *S. pneumoniae* by the BC-GP panel be confirmed by Gram stain plus bile solubility or other test methods. Careful reporting of all *S. pneumoniae* identified by BC-GP as *Streptococcus* spp. when atypical Gram stain morphology was present prevented incorrect reporting in cases of viridans group streptococci. The authors further suggested that incorporating a *S. pneumoniae* antigen test on blood culture broth may complement BC-GP to permit earlier reporting of results for *S. pneumoniae*.

Overall, the results from this five-year retrospective study confirmed that the BC-GP test has outstanding performance, thus allowing clinicians to de-escalate antimicrobial therapy in the absence of detection of a *mecA* and *vanA/B* gene with confidence.

Beckman and coworkers described a study which evaluated the performance of BC-GP on 2115 positive pediatric blood culture specimens (Beckman et al., 2019). The results of BC-GP were compared to the results from conventional culture and susceptibility testing. Of the positive cultures, 1503 were gram-positive by Gram stain and 1231 grew single isolates that were detectable by the BC-GP assay. Out of the 1231 single isolate cultures, 899 were accurately identified to the species level, 282 were identified to the genus, and 50 were not detected by BC-GP. Except for seven organisms, all organisms detected by BC-GP in monomicrobial cultures were also identified as the same organism by traditional methods. There were no differences in the overall agreement for detection of *Enterococcus*, *Streptococcus*, *Staphylococcus* spp., *S. aureus*, *E. faecium*, and *S. agalactiae*. *E. faecalis* was correctly identified in all but 3 of 88 and *S. pyogenes* in all but 1 of 25. The seven isolates with results different than the culture method were correctly identified as *Streptococcus*, but incorrectly as *S. pneumoniae*, instead of *S. mitis/oralis*. Polymicrobial growth of gram-positive organisms was found in 112 cultures. Of these, BC-GP correctly identified all organisms present that were included in the panel in 77 cultures. Exceptional overall agreement was also observed for antimicrobial resistance markers, with only five samples (1%) showing discordant results. For almost all isolates, the BC-GP assay result was as useful as conventional culture identification, and the detection of antimicrobial resistance markers was highly accurate. Therefore, the data acquired from BC-GP can be confidently used by clinicians to support antimicrobial selection. Based on review of 20 cases where results of BC-GP disagreed with that of the conventional methods, the authors found little to no impact or effect clinically on patients or their management, which was likely due to the maintenance of empiric therapy, and no patients were placed at an increased risk of harm.

### The Luminex^®^ VERIGENE^®^ Gram-Negative Blood Culture Test

VERIGENE BC-GN test performance was determined in a clinical study conducted at twelve institutions across the U.S. (Luminex, 2020a). Of the 1434 specimens enrolled in the trial, 73 were invalid on initial testing (5.1%) but gave a valid result upon retesting (final invalid rate of 2.3%). A total of 1412 specimens were included for evaluation, including 876 prospectively collected specimens (604 fresh and 175 frozen), 239 pre-selected frozen specimens, and 297 simulated frozen specimens. The results of BC-GN were compared to reference culture followed by biochemical identification. Results for bacterial resistance markers were compared to results of PCR amplification, followed by bidirectional sequencing. The PPA, NPA, and two-sided 95% CI observed for detection of each organism and resistance marker are shown in **Table 4**. Overall, the PPA was ≥92.2% and the NPA was ≥99.4%. *Serratia marcescens* is not an IVD-cleared target in the U.S. but a separate study conducted at a single site showed a PPA of 88.5% (23/26) (Luminex, 2020b).

**Table 4:** Summary of clinical performance for the VERIGENE Gram-Negative Blood Culture test.

Target	Fresh		Frozen		Pre-selected		Simulated		Total		
	PPA^a^N ^b^(95% CI^c^)	NPA^d^N (95% CI)	PPAN (95% CI)	NPAN (95% CI)	PPAN (95% CI)	NPAN (95% CI)	PPAN (95% CI)	NPAN (95% CI)	PPAN (95% CI)		NPAN (95% CI)
*Escherichia coli*	100% 283/283 (98.7-100)	99.1% 318/321 (97.3-99.8)	99.3% 142/143 (96.2-100)	99.2% 128/129 (95.8-100)	100% 42/42 (91.6-100)	99.5% 196/197 (97.2-100)	100% 50/50 (92.9-100)	100% 247/247 (98.5-100)	99.8% 517/518 (98.9-100)		99.4% 889/894 (98.7-99.8)
*Klebsiella pneumoniae*	88.0% 88/100 (80.0-93.6)	100% 504/504 (99.3-100)	87.0% 40/46 (73.1-95.1)	100% 226/226 (98.4-100)	94.7% 36/38 (82.3-99.4)	100% 201/201 (98.2-100)	99.2% 121/122 (95.5-100)	100% 175/175 (97.9-100)	93.1% 285/306 (89.7-95.7)		100% 1106/1106 (99.7-100)
*Klebsiella oxytoca*	95.7& 22/23 (78.1-99.9)	98.2% 576/581 (97.9-100)	100% 9/9 (66.4-100)	99.6% 262/263 (97.9-100)	92.6% 25/27 (75.7-99.1)	100% 212/212 (98.3-100)	60.0% 3/5 (14.7-94.7)	100% 292/292 (98.7-100)	92.2% 59/64 (82.7-97.4)		99.6% 1342/1348 (99.0-99.8)
*Pseudomonas aeruginosa*	97.1% 67/69 (89.9-99.7)	100% 535/535 (99.3-100)	91.7% 11/12 (61.5-99.8)	100% 260/260 (98.6-100)	100% 19/19 (82.4-100)	100% 220/220 (98.3-100)	100% 27/27 (87.2-100)	100% 270/270 (98.6-100)	97.6% 124/127 (93.3-99.5)		100% 1285/1285 (99.7-100)
*Serratia marcescens* ^e^	–	–	–	–	–	–	–	–	88.5% 23/26 (69.9-97.6)		99.9% 294/297 (97.1-99.8)
*Acinetobacter* spp.	100% 12/12 (73.5-100)	100% 592/592 (99.4-100)	50% 1/2 (1.3-98.7)	100% 270/270 (98.6-100)	100% 15/15 (78.2-100)	99.6% 223/224 (97.6-100)	100% 27/27 (87.2-100)	100% 270/270 (98.6-100)	98.2% 55/56 (90.5-100)		99.9% 1355/1356 (99.6-100)
*Citrobacter* spp.	100% 5/5 (47.8-100)	99.8% 598/599 (99.1-100)	100% 1/1 (2.5-100)	100% 271/271 (98.7-100)	100% 13/13 (75.3-100)	100% 226/226 (98.4-100)	100% 30/30 (88.4-100)	100% 267/267 (98.6-100)	100% 49/49 (92.8-100)		99.9% 1362/1363 (99.6-100)
*Enterobacter* spp.	95.6% 43/45 (84.9-99.5)	100% 559/559 (99.3-100)	95.2% 20/21 (76.2-99.9)	98.4% 247/251 (96.0-99.6)	100% 29/29 (88.1-100)	98.1% 206/210 (95.2-99.5)	100% 28/28 (87.7-100)	100% 269/269 (98.6-100)	97.6% 120/123 (93.0-99.5)		99.4% 1281/1289 (98.8-99.7)
*Proteus* spp.	100% 20/20 (83.2-100)	100% 584/584 (99.4-100)	100% 12/12 (73.5-100)	100% 260/260 (98.6-100)	100% 24/24 (85.8-100)	99.5% 214/215 (97.4-100)	100% 2/2 (15.8-100)	100% 295/295 (98.8-100)	100% 58/58 (93.8-100)		99.9% 1353/1354 (99.6-100)
CTX-M	97.5% 39/40 (86.8-99.9)	100% 497/497 (99.3-100)	91.2% 11/12 (61.5-99.8)	100% 224/224 (98.4-100)	100% 3/3 (29.2-100)	100% 203/203 (98.2-100)	100% 98/98 (96.3-100)	99.5% 188/189 (97.1-100)	98.7% 151/153 (95.4-99.8)		99.9% 1112/1113 (99.5-100)
IMP	–	100% 537/537 (99.3-100)	–	100% 236/236 (98.5-100)	–	100% 206/206 (98.2-100)	100% 48/48 (92.6-100)	100% 239/239 (98.5-100)	100% 48/48 (92.6-100)		100% 1218/1218 (99.7-100)
KPC	100% 2/2 (15.8-100)	100% 535/535 (99.3-100)	100% 1/1 (2.5-100)	100% 235/235 (985-100)	–	100% 206/206 (98.2-100)	100% 48/48 (92.6-100)	100% 239/239 (98.5-100)	100% 51/51 (93.1-100)		100% 1215/1215 (99.7-100)
NDM	100% 1/1 (2.5-100)	100% 536/536 (99.3-100)	–	100% 236/236 (98.5-100)	–	100% 206/206 (98.2-100)	100% 40/40 (91.2-100)	100% 247/247 (98.5-100)	100% 41/41 (91.4-100)		100% 1225/1225 (99.7-100)
OXA	100% 5/5 (47.8-100)	100% 532/532 (99.3-100)	–	100% 236/236 (98.5-100)	50.0% 2/4 (6.8-93.2)	100% 202/202 (98.2-100)	98.3% 54/55 (90.8-100)	99.6% 231/232 (97.6-100)	95.3% 61/64 (86.9-99.0)		99.9% 1201/1202 (99.5-100)
VIM	–	100% 537/537 (99.3-100)	–	100% 236/236 (98.5-100)	–	100% 206/206 (98.2-100)	100% 41/41 (91.4-100)	100% 246/246 (98.5-100)	100% 41/41 (91.4-100)		100% 1225/1225 (99.7-100)

a PPA, Positive Percent Agreement.

b N, number.

c 95% CI, 95% Confidence Interval.

d NPA, Negative Percent Agreement.

e Serratia marcescens is not IVD-cleared in the United States. Data were collected in a separate study conducted at a single external clinical site.

The data above were included in a 2015 publication by Ledeboer et al. which described the results from a total of 1,847 blood cultures positive for gram-negative organisms (Ledeboer et al., 2015). The full sample set included 729 fresh, prospective, 781 frozen (prospective or retrospective), and 337 simulated cultures, and represented 7 types of aerobic culture media. Among monomicrobial cultures, the PPA of BC-GN with the reference result ranged from 92.9%-100% for the organism identification targets. The NPA for organism identification ranged from 99.5% to 99.9%. The authors noted that 25/26 cultures containing *Klebsiella pneumoniae* reported as “not detected” by BC-GN were identified as *Klebsiella variicola* by subsequent sequence analysis. The PPA for identification of resistance determinants was ≥94.3% and the NPA was >99.9%. The overall PPA and NPA for the eight organism identification targets was 97.9% and 99.7%, respectively. In polymicrobial specimens (with gram-negative appearance on Gram stain), the BC-GN assay accurately identified a minimum of one organism in 95.4% of the cultures and identified all organisms correctly in 54.5%. The prevalence of genetic resistance determinants was low in the prospective sample set (0.0% to 0.6%), except for CTX-M (5.0%), so performance for these targets was assessed using simulated specimens. The PPA for KPC, VIM, and IMP was 100%. The PPA was 98.9% for CTX-M, 96.2% for NDM, and 94.3% for OXA. The NPA was 100% for all but the simulated OXA specimens (99.6%). Overall, the PPA and NPA were 98.3% and 99.9% for the six genetic resistance markers.

The authors stated that a unique advantage of the BC-GN assay versus other rapid identification methods (e.g., MALDI-TOF MS and peptide nucleic acid fluorescence *in situ* hybridization [PNA FISH]) is the capacity to identify six genetic resistance markers associated with various classes of β-lactam antibiotics. While the frequency of carbapenemases found in this study was low, epidemiology and surveillance data has shown the prevalence steadily increasing over time (Nordmann et al., 2011; van Duin and Doi, 2017). The authors found that the targets included in the BC-GN test enabled identification of the infecting organism in about 90% of the prospective blood culture specimens which contained gram-negative bacilli. They noted that some relatively common gram-negative pathogens, such as *Serratia* spp., *Stenotrophomonas maltophilia*, and non-aeruginosa *Pseudomonas* spp., accounted for 4.4% of the positive cultures but are not included in BC-GN. Additionally, since *K. variicola* may account for up to 10% of all *Klebsiella* spp. isolated from clinical specimens, additional sequencing or biochemical testing may be desirable in the event of a *K. pneumoniae* blood culture identification that was not detected by BC-GN.

As described in the study above, BC-GN identified at least one pathogen in the majority of polymicrobial cultures but could only identify all pathogens present in about half of these cases. Identification of all pathogens present in polymicrobial cultures may be particularly challenging for a technology designed for direct analysis of specimens (e.g., molecular, MALDI-TOF MS, etc.). Use of these tests, such as BC-GN, could be limited in clinical settings due to reduced sensitivity in polymicrobial infections and concomitant concerns for unsuitable antibiotic modification as a result. Although Gram stain is used to confirm the morphology of the organism for appropriate test selection, multiple organisms may have similar appearance by Gram stain. Additionally, when gram-negative organisms are detected in multiple bottles in a set, typically one bottle is prepared for testing and thus the possibility exists that the gram-negative organisms observed in may be different and not all tested using BC-GN. To investigate this potential issue, Claeys and colleagues conducted a retrospective review of 1003 blood culture sets from 2 institutions and found the incidence of missed gram-negative identification to be infrequent at <4% (Claeys et al., 2018a). Since the patient data were not reviewed, a hypothetical analysis was conducted based on assumption that all patients were treated empirically with a gram-negative antibiotic (as directed by their institutional guidelines). Using this analysis, the investigators found that of the missed gram-negative identifications, an adverse impact on patient management could have potentially occurred in <2% of cases. The authors state that it is essential for laboratories to be cognizant of the possibility to miss detection of organisms in polymicrobial cultures so that these cases do not negatively affect stewardship efforts. Even with these known limitations, it is appropriate to trust the BC-GN result in combination with clinical judgment, considering the importance of limiting broad-spectrum antibiotic usage.

While rapid detection of genetic resistance markers in gram-negative BSIs allows quick optimization of empiric antibiotic therapy, how to respond when none are detected is unclear, given the complex nature of gram-negative resistance and the safety of de-escalation in this setting may be unknown. Pogue et al. analyzed the results from 1046 gram-negative blood culture isolates analyzed by the BC-GN test to determine the NPVs of resistance marker absence to predict susceptibility in specific drug-bug combinations at two different sites (Pogue et al., 2018). Except for *Pseudomonas aeruginosa*, the absence of resistance genes detected by BC-GN mostly predicted susceptibility to target antibiotics at both institutions. NPVs for ceftriaxone susceptibility in *Escherichia coli* and *K. pneumoniae* in the absence of either a carbapenemase gene or CTX-M were 93%-94% and 98%, respectively. Similar results were seen for other target bug-drug scenarios, with NPVs of 94% to 100% at both sites, except for *P. aeruginosa*, where the NPVs were poor. This is most likely related to the complexity of antibiotic resistance in this organism. For all other organisms, susceptibility was mainly predicted by the presence or absence of the resistance determinants included in BC-GN. The results of this study showed that clinicians at these institutions could have confidence in antibiotic de-escalation when no resistance determinants are detected and treatment decisions can be based on the results of the BC-GN test (for both escalation and de-escalation). The authors proposed that the methodology they describe could serve as a blueprint for antimicrobial stewardship to assess how BC-GN performs in these scenarios and optimize management of gram-negative bacteremia in their patients.

## Clinical and Economic Impact

### Gram Positive Bloodstream Infections

Enterococci are a significant cause of BSI in hospitalized patients and there are few antimicrobial treatments due to numerous resistance mechanisms. Sango and coworkers evaluated the impact of rapid organism identification and resistance detection with the BC-GP assay on the clinical and economic outcomes for 74 patients with bacteremia due to *Enterococcus* (Sango et al., 2013). This pre/postintervention study compared non-equivalent groups of inpatients with enterococcal bacteremia over 13 months in a quasi-experimental design. Prior to implementation of BC-GP testing, Gram stain results from positive cultures were called to the patient care area and identification and susceptibility results were reported when available. After the BC-GP assay was implemented, an infectious disease and/or critical care pharmacist was notified of the BC-GP results and appropriate antibiotics were recommended. The preintervention group included 46 patients vs. 28 in the postintervention group, with vancomycin-resistant *Enterococcus* (VRE) bacteremia occurring in 37% and 50%, respectively, which was not significantly different (*P* = 0.33).

The mean time to appropriate antimicrobial therapy was decreased by 23.4 hours in the postintervention group compared to the preintervention group (*P* = 0.0054). The mean time to appropriate therapy for patients with a vancomycin-susceptible *Enterococcus* (VSE) BSI was 18.6 hours in the postintervention group compared to 40.2 hours preintervention, but this decrease was not significant (*P* = 0.1145). However, for patients with VRE bacteremia, the mean time to appropriate antimicrobial therapy was significantly shorter by 31.1 hours in the postintervention group (*P*<0.0001). Also, in the postintervention group, the length of hospital stay was significantly shorter by 21.7 days (*P* = 0.0484) and mean hospital costs were $60,729 lower (*P* = 0.02) than in the preintervention group. There was not a significant difference in the mortality rates in the two groups. The study shows that microarray technology, such as the BC-GP assay, with support from pharmacy and the microbiology laboratory, can substantially decrease the time to suitable antimicrobial therapy, the length of hospital stay, and hospital costs.

A study reported by Box et al. also demonstrated the clinical and economic impact of rapid diagnostics for BSI (Box et al., 2015). The investigators evaluated the effect of implementing the BC-GP test with real-time support from the antibiotic stewardship team (AST) in a community hospital setting. This pre/post, quasi-experimental study was conducted at the five hospitals in the Scripps Healthcare system. Rapid diagnostic testing using BC-GP was performed at the central laboratory for 12 hours each day and pharmacists notified physicians of the results and assisted with antibiotic modifications. Primary outcomes measured were the average time to targeted antibiotic therapy and the difference in antibiotic duration for contaminant organisms. Secondary end points measured included mortality, length of hospital stay, pharmacy costs, and overall hospitalization costs. Of 167 adult patients with a gram-positive BSI, 103 patients (admitted in 2011) were included in the preintervention group and 64 patients (admitted in 2014) were in the postintervention group.

Implementation of rapid identification using BC-GP in combination with AST intervention improved the average time to targeted antibiotic therapy from 61.1 hours to 35.4 hours (*P*<0.001) and decreased the average duration of antibiotic therapy for blood culture contaminants from 42.3 hours to 24.5 hrs (*P* = 0.03). The median length of hospital stay was decreased from 9.1 days to 7.2 days postintervention (*P* = 0.04), and the overall median hospitalization costs were decreased from $17,530 to $10,290 (*P* =0.04). Median pharmacy costs were similar ($822 vs. $425, *P* = 0.11), as were mortality rates at 9.1% and 9.2% (*P* = 0.98). Rapid identification of gram-positive blood culture isolates with AST intervention has a positive impact on patient outcomes with less time to targeted antibiotic therapy, less time on unnecessary antibiotic therapy, a shorter length of hospital stay, and lower overall hospital costs.

Investigators at Children’s Hospital Los Angeles conducted a study to determine the clinical and economic impact of the BC-GP assay on the diagnosis of gram-positive BSIs in pediatric patients (Felsenstein et al., 2016). Data on time to optimization of antibiotics, length of hospitalization, and hospital cost were collected prospectively following implementation of BC-GP testing and were compared with matching preimplementation data. A total of 440 gram-positive BSI episodes from 383 patients were included, with 219 postimplementation and 221 preimplementation. Polymicrobial infections were included and the pathogens identified were considered when determining appropriate antimicrobial treatment. A pediatric infectious diseases physician and a pharmacist separately assessed the time to antimicrobial optimization, de-escalation, and discontinuation, and the clinical suitability of these decisions. Agreement between the independent assessments was high, and discrepancies were resolved by including another pediatric infectious diseases physician in a third assessment. The overall hospital costs were calculated based on all direct costs incurred within all services during hospitalization, which included lodging, meals, medication, consults, radiology, and laboratory costs.

The time to antimicrobial optimization was shortened by 12.5 hours overall (*P* = 0.006), and by 11.9 hours specifically for *S. aureus* (*P* = 0.005) postimplementation. For patients with likely blood-culture contamination by coagulase-negative staphylococci (CoNS), the duration of antibiotics was decreased by 36.9 hours (*P*<0.001). The median length of hospital stay for patients admitted to a general ward was 1.5 days shorter (*P* = 0.04), and the median hospital cost was $3,757 less (*P* = 0.03) after implementation. For *S. aureus* BSIs specifically, the median length of hospital stay and overall costs were decreased by 5.6 days (*P* = 0.01) and $13,341 (*P* = 0.03), respectively. Mortality within one month of BSI diagnosis was low, trending toward decreased mortality after implementation, but this difference was not significant (5.7% preimplementation vs. 2.6% postimplementation, *P* = 0.28). Admission rate to the intensive care unit (ICU) was also not significantly different between the two time periods (40.7% vs. 48.1%, *P* = 0.24).

Eby and colleagues reported the effect of implementing BC-GP testing together with a triggered, mandatory infectious diseases (ID) consultation, to improve management and outcomes of patients with *S. aureus* bacteremia (Eby et al., 2018). Prior to implementation, *S. aureus* BSI was identified by conventional culture and the results sent to ASP pharmacists. After implementation, *S. aureus* BSI in adult inpatients was identified by BC-GP and the results were paged directly to ID fellows, with immediate initiation of a consultation. The ASP supported management after the initial consultation. Retrospective, pre/postintervention analysis was performed on 106 preintervention and 120 postintervention subjects. The time to a consultation after positive blood culture notification was decreased by 26.0 hours (95% CI, 45.1 hours to 7.1 hours, P < 0.001) postintervention as compared to preintervention. The time to starting targeted antibiotic treatment decreased by 21.2 hours on average (95% CI, 31.4 hours to 11.0 hours, P < 0.001) and the time to targeted antibiotics for methicillin-sensitive *S. aureus* (MSSA) decreased by an average of 40.7 hours (95% CI, 58.0 hours to 23.5 hours, P < 0.001). BC-GP plus intervention also resulted in lower mortality in the hospital (13.2% to 5.8%, P = .047) and a lower 30-day mortality (17.9% to 8.3%, P = .025). A more efficient response to *S. aureus* BSI with BC-GP testing was associated with improved care and outcomes for these patients.

### Gram Negative Bloodstream Infections

Walker et al. conducted a retrospective review of gram-negative BSI in hospitalized patients over 6-months before (98 subjects) and 6-months after (97 subjects) implementing the BC-GN test for detection of gram-negative organisms in positive blood cultures (Walker et al., 2016). Antimicrobial stewardship practices were not changed. Patient demographics, the time to organism identification, the time to effective antimicrobial therapy, and some additional clinical parameters were compared. There was no significant difference between the two groups with respect to comorbidities, cause of bacteremia, or admissions to the ICU, as well as use of immunosuppressive therapies, neutropenia, or bacteremia that was due to organisms that were multi-drug resistant. The BC-GN assay resulted an identification in 87% of gram-negative cultures and was correct in 98% (95/97) of the cases, as compared to results from conventional culture. Organism identification was completed faster after BC-GN implementation (mean = 10.9 hours vs. 37.9 hours, *P*<0.001). Length of ICU stay was 4.2 days shorter (P = 0.033) and the 30-day mortality was 11.1% lower (P = 0.037). The mortality associated with multidrug-resistant organisms occurred in 1 of 8 cases postintervention vs. 12 of 19 cases in the preintervention group (P = 0.033). Implementation of effective therapy was significantly faster in the postintervention cases for extended-spectrum beta-lactamase (ESBL)-producing organisms (P = 0.049), but not significantly faster overall (P = 0.12). The authors estimated the cost per BC-GN test to be $99 but the average net savings per ICU patient would be $11,661. The data show the BC-GN assay was a valuable tool for early identification of gram-negative organisms BSIs and had a significant impact on patient care, especially when genetic resistance markers were detected.

The effect of BC-GN together with an antimicrobial intervention on clinical outcomes was also investigated by Rivard and co-workers in a large pre/post implementation study conducted at The Cleveland Clinic on 877 patients (456 preimplementation and 421 postimplementation) with a gram-negative BSI (Rivard et al., 2017). The main objective was to compare the time from Gram stain to an antimicrobial change. Then the time from Gram stain to effective treatment, the in-hospital mortality rate, and the length of hospital stay were also compared. Prior to implementation of BC-GN, microbiological services were not centralized and blood cultures were processed at regional facilities during two shifts, whereas blood cultures at the academic medical center were processed 24 hours per day. After implementation of BC-GN, an active ASP was utilized with centralized microbiological processing. Both groups had daily audits and feedback on antimicrobial therapy by the ASP pharmacists to identify possible optimization based on the results. An institutional guideline was developed to guide therapy recommendations.

The number and types of antimicrobial changes were similar between the groups. The median time from Gram stain to an antimicrobial change was significantly less in the postimplementation group at 28.6 hours, compared to 44.1 hours in the preimplementation group (*P* = 0.004). Time to effective therapy for patients on inappropriate therapy at the time of Gram stain was reduced in the postimplementation group (8.8 hours vs. 24.5 hours, *P* = 0.034). Additionally, the median length of hospital stay was also decreased in the postimplementation group to 7 days compared to 9 days preimplementation (*P* = 0.001). The in-hospital mortality rate was similar at 11.6% (preimplementation) vs. 11.4% (postimplementation), *P* = 0.87. This evaluation supports the incorporation of rapid diagnostic technologies, such as the BC-GN test, into antimicrobial stewardship programs.

A study reported by Claeys et al. investigated the possible impact of an antibiotic treatment algorithm, driven by stewardship and incorporating rapid testing by the BC-GN test into the management of gram-negative bacteremia (Claeys et al., 2018b). This retrospective, observational study of adult patients included a total of 188 patients with gram-negative BSI, of which 144 (76.5%) were positive for organisms targeted by BC-GN. The evaluation employed an AMS-driven treatment algorithm based on data from institution-specific antibiograms with an evidence-based practice for managing drug-resistant gram-negative organisms.

Susceptibility *in vitro* was higher overall with antibiotics recommended by the algorithm as compared with the standard practice for isolates that could be assessed (92.1% versus 77.8%). Among the 144 patients with BC-GN panel organisms, there was a modest level of agreement between reviewers on the suitability of the standard antibiotics (κ = 0.735) but a strong level of agreement for those recommended by the algorithm (κ = 0.855). The algorithm proposed would have allowed 88.4% of cases to receive appropriate antibiotic therapy versus only 78.1% by the standard of care antibiotics (*P* = .014). The AMS treatment algorithm would have led to a 14.4% appropriate de-escalation, a 4.8% inappropriate de-escalation, a 5.3% appropriate escalation, and a 16.0% unnecessary escalation. The presence of a polymicrobial gram-negative BSI, a central line source for a gram-negative BSI, *Acinetobacter* spp., *Enterobacter* spp., and/or an OXA+ resistance determinant was associated with inappropriate recommendations by the AMS treatment algorithm (*P* <.05). The authors concluded that the ability to develop a treatment algorithm based on institutional antibiogram data and combined with evidence-based practices and BC-GN results, had the possibility to improve the percentage of gram-negative BSI patients receiving timely appropriate antimicrobial therapy.

## Conclusion

Technological advances in molecular methods have revolutionized the approach to diagnosis of microbial pathogens in the microbiology laboratory. As these sophisticated methods have become faster, more automated, and simpler to use, they can be easily implemented into the laboratory workflow and have become an integral part of the routine testing repertoire in most diagnostic laboratories.

Although conventional culture techniques remain the foundation for diagnosis of BSIs in most microbiology laboratories, a move toward novel technologies that can identify pathogens and resistance markers directly from blood culture is critically important to optimize treatment and improve patient outcomes. Fortunately, several of these technologies have become available in the last decade to aid in diagnosis of BSIs. Rapid molecular tests, such as the VERIGENE Blood Culture Nucleic Acid Tests, which allow direct identification of bacteria and genetic resistance markers from positive blood cultures, have been shown to perform with extremely high sensitivity and specificity.

It should be noted that there are some limitations to the use of molecular tests for diagnosis of bloodstream pathogens as such tests can only identify those pathogens included in the panels (Lamy et al., 2020). In addition, detection of genetic resistance markers does not preclude phenotypic resistance due to other mechanisms. Identification of the pathogens present in a polymicrobial culture is also difficult and could impact the clinical use of tests such as BC-GN, as a lower sensitivity when multiple pathogens are present could limit the ability to modify antibiotic treatment. And, as reported for BC-GP, differentiation of *S. pneumoniae* from *S. mitis/oralis* is particularly challenging for a molecular test, since the organisms share a 99% sequence homology. Therefore, it would be prudent to confirm *S. pneumoniae* results with additional tests and to refrain from reporting it to the species level.

Altogether, the studies reported in the literature and reviewed here prove the clinical utility of such testing and clearly demonstrate the positive impact on patient outcomes and reduction of overall healthcare costs. Furthermore, the data described here builds the case for these rapid molecular methods as key components in the approach to both diagnostic and antimicrobial stewardship in the healthcare system.

## Author Contributions

SAD prepared this manuscript. CG and SD prepared the outline, participated in selection of articles to include, and reviewed/edited the manuscript. Final selection of articles to include was conducted by SAD and CG. All authors contributed to the article and approved the submitted version.

## Conflict of Interest

The authors are employees of Luminex, A DiaSorin Company who is the manufacturer of the tests reviewed in this manuscript. 

## Publisher’s Note

All claims expressed in this article are solely those of the authors and do not necessarily represent those of their affiliated organizations, or those of the publisher, the editors and the reviewers. Any product that may be evaluated in this article, or claim that may be made by its manufacturer, is not guaranteed or endorsed by the publisher.
